# Supplementary material for: In Vitro Confirmation of Siramesine as a Novel Antifungal Agent with In Silico Lead Proposals of Structurally Related Antifungals
Source: Molecules. 2021 Jun 8;26(12):3504. doi: 10.3390/molecules26123504 (PMC8230181; doi:10.3390/molecules26123504)
Supplement: Supplementary file 1 [file molecules-26-03504-s001.zip › Supplementary files/Table S1.docx]

Table S2. Cross-validated prediction of pKi on the training set

| Comp | Exp pKi | Gold pKi | Adt pKi | Comp | Exp pKi | Gold pKi | Adt pKi | Comp | Exp pKi | Gold pKi | Adt pKi |
| --- | --- | --- | --- | --- | --- | --- | --- | --- | --- | --- | --- |
| **RC-33** | 9.16 | 8.78 | 8.83 | **29** | 6.34 | 7.28 | 7.40 | **56** | 8.82 | 8.85 | 8.59 |
| **2** | 7.6 | 7.14 | 7.30 | **30** | 7.85 | 7.31 | 7.41 | **57** | 8.22 | 8.69 | 8.72 |
| **4** | 6.99 | 6.45 | 7.11 | **31** | 7.04 | 6.95 | 7.01 | **59** | 8.27 | 8.17 | 8.07 |
| **5** | 7.7 | 7.72 | 8.02 | **32** | 8.36 | 8.09 | 8.22 | **60** | 6.94 | 7.45 | 7.01 |
| **6** | 7.67 | 7.95 | 8.10 | **34** | 7.64 | 8.36 | 8.44 | **61** | 7.7 | 8.00 | 7.98 |
| **7** | 8.85 | 8.16 | 8.16 | **35** | 8.15 | 8.32 | 8.38 | **62** | 8.46 | 8.47 | 8.51 |
| **9** | 6.09 | 6.87 | 6.83 | **36** | 8.97 | 7.73 | 7.75 | **64** | 6.62 | 6.68 | 6.54 |
| **10** | 6 | 5.85 | 5.92 | **37** | 8.38 | 7.91 | 7.83 | **65** | 7.44 | 7.40 | 7.20 |
| **11** | 7.33 | 7.42 | 7.59 | **39** | 8.02 | 7.56 | 7.63 | **66** | 8.54 | 8.24 | 8.18 |
| **12** | 8.71 | 8.33 | 8.10 | **40** | 7.94 | 7.68 | 7.67 | **67** | 6.86 | 6.89 | 6.76 |
| **14** | 8.64 | 8.44 | 8.61 | **41** | 6 | 6.75 | 6.88 | **69** | 7.34 | 7.92 | 7.98 |
| **15** | 8.99 | 8.66 | 8.77 | **42** | 6 | 6.16 | 6.35 | **70** | 8.54 | 8.37 | 8.14 |
| **16** | 8.22 | 8.17 | 7.72 | **44** | 6 | 6.14 | 5.94 | **71** | 8.52 | 8.33 | 8.18 |
| **17** | 8.62 | 7.70 | 7.80 | **45** | 6 | 7.07 | 6.72 | **72** | 7.07 | 6.94 | 7.06 |
| **19** | 8.2 | 8.60 | 8.36 | **46** | 6 | 6.44 | 6.80 | **74** | 7.96 | 7.91 | 7.91 |
| **20** | 7.86 | 8.37 | 8.24 | **47** | 6 | 5.45 | 5.11 | **75** | 7.57 | 7.68 | 7.38 |
| **21** | 7.04 | 7.78 | 7.70 | **49** | 6 | 5.47 | 5.48 | **76** | 7.4 | 7.58 | 7.45 |
| **22** | 8.28 | 7.51 | 7.41 | **50** | 7.41 | 8.12 | 8.02 | **77** | 7.15 | 7.42 | 7.56 |
| **24** | 8.24 | 8.89 | 8.91 | **51** | 8.33 | 8.03 | 7.96 | **79** | 7.46 | 8.05 | 8.16 |
| **25** | 8.64 | 8.87 | 8.62 | **52** | 6.69 | 7.37 | 7.34 | **80** | 7.89 | 7.56 | 7.94 |
| **26** | 7.98 | 7.80 | 7.71 | **54** | 7.29 | 7.61 | 7.70 | 4-IBP | 8.59 | 7.06 | 7.02 |
| **27** | 9.01 | 8.11 | 8.30 | **55** | 7.6 | 7.55 | 7.55 | PD144418 | 8.37 | 8.57 | 8.76 |
