# Supplementary material for: In Vitro Confirmation of Siramesine as a Novel Antifungal Agent with In Silico Lead Proposals of Structurally Related Antifungals
Source: Molecules. 2021 Jun 8;26(12):3504. doi: 10.3390/molecules26123504 (PMC8230181; doi:10.3390/molecules26123504)
Supplement: Supplementary file 1 [file molecules-26-03504-s001.zip › Supplementary files/Supplementary_data_files.zip!/Supplementary_data_files/UVE_PLS_code_for Sigma1.doc]

A1<-read.table("Sigma1_**83_ref_sigma1recref_UPDATED_from_GOLD_2019_1D_2D_3D_1836_var**.txt")

B2<-c(9.16, 7.6, 7.41, 6.99, 7.7, 7.67, 8.85, 6, 6.09, 6, 7.33, 8.71, 7.72, 8.64, 8.99, 8.22, 8.62, 8.1, 8.2, 7.86, 7.04, 8.28, 8.27, 8.24, 8.64, 7.98, 9.01, 9.07, 6.34, 7.85, 7.04, 8.36, 8.89, 7.64, 8.15, 8.97, 8.38, 8.11, 8.02, 7.94, 6, 6, 6, 6, 6, 6, 6, 6, 6, 7.41, 8.33, 6.69, 7.2, 7.29, 7.6, 8.82, 8.22, 8.16, 8.27, 6.94, 7.7, 8.46, 7.12, 6.62, 7.44, 8.54, 6.86, 6.37, 7.34, 8.54, 8.52, 7.07, 8, 7.96, 7.57, 7.4, 7.15, 7.21, 7.46, 7.89, 8.59, 8.37, 7.66) # These are experimental pKi values (exactly following the labels)

pon<-150

ncomp<-20 #novo

Fe1<-data.matrix(A1)

F<-Fe1

Sys.time()

sekta<-3

V<-data.frame(F=I(F),**B2=B2**)

Vtest<-seq(sekta,length(B2),by=5)

Vrum<-seq(1,length(B2),by=1)

Vtrain<-Vrum[-Vtest]

B1<-V$B2[Vtrain]

Fe<-V$F[Vtrain,]

broelim<-0

for (i in 1:ncol(Fe)) {

if (length(unique(Fe[,i]))<3) {

broelim<-broelim+1

if (broelim==1)

vectvoid<-c(i)

else

vectvoid<-cbind(vectvoid,i)}

}

for (i in length(vectvoid):1) {

Fe<-Fe[,-vectvoid[i]]

F<-F[,-vectvoid[i]]}

A3<-read.table("Sigma1_siramesine_1836_vector_Gold.txt")

A4<-read.table("Sigma1_59_S_siramesine_similar_comp_from_GOLD_1D_2D_3D_flex_1836_var.txt")

A5<-rbind(A3,A4)

Fe5<-data.matrix(A5)

for (i in length(vectvoid):1) {

Fe5<-Fe5[,-vectvoid[i]]}

Bvarr<-c()

minn<-array(ncol(F))

maxx<-array(ncol(F))

for (i in 1:ncol(F)) {

minn[i]<-min(F[,i])

maxx[i]<-max(F[,i])

bro<-0

for (j in 1:nrow(Fe5)) {

if ((Fe5[j,i]>=minn[i])&&(Fe5[j,i]<=maxx[i]))

bro<-bro+1}

if (bro==nrow(Fe5))

Bvarr<-cbind(Bvarr,c(i))}

Fe<-Fe[,Bvarr]

F<-F[,Bvarr]

Fe5<-Fe5[,Bvarr]

meanvect<-array(ncol(Fe))

sdvect<- array(ncol(Fe))

for (i in 1:ncol(Fe)) {

meanvect[i]<-mean(Fe[,i])

sdvect[i]<-sd(Fe[,i])}

Fe<-scale(Fe)

for (i in 1:ncol(F)) {

F[,i]<- (F[,i]- meanvect[i])/sdvect[i] }

for (i in 1:ncol(Fe5)) {

Fe5[,i]<- (Fe5[,i]- meanvect[i])/sdvect[i] }

V<-data.frame(F=I(F),**B2=B2**)

Vtest<-seq(sekta,length(B2),by=5)

Vrum<-seq(1,length(B2),by=1)

Vtrain<-Vrum[-Vtest]

B1<-V$B2[Vtrain]

Fe<-V$F[Vtrain,]

RMSECV<-array(ncomp)

PRESS<-array(ncomp)

Btest<-array(nrow(Fe))

xtrain<-Fe

xtest<-matrix(rep(NA,1*ncol(Fe)),nrow=1)

B.pred<-matrix(rep(NA, ncomp*nrow(Fe)),nrow=ncomp)

for (i in 1:ncomp) {

PRESS[i]<-0}

for (m in 1:nrow(Fe)) {

xtest<-Fe[m,]

Btest[m]<-B1[m]

xtrain<-Fe[-m,]

Btrain<-B1[-m]

Ftrain<-t(t(Btrain)-mean(Btrain))

Etrain<-t(t(xtrain)-colMeans(xtrain))

Xb<-xtest- colMeans(xtrain)

lj<-Ftrain

T<-matrix(rep(NA, nrow(Etrain)*1),nrow=nrow(Etrain))

W<-matrix(rep(NA, ncol(Etrain)*1),nrow=ncol(Etrain))

P<-matrix(rep(NA, ncol(Etrain)*1),nrow=ncol(Etrain))

Q<-array(1)

for (i in 1:ncomp) {

S<-t(Etrain)%*%Ftrain

qp<-svd(S)

if (i==1) {

W[,i]<-qp$u

T[,i]<-Etrain%*%W[,i] }

else {

W<-cbind(W,qp$u)

T<-cbind(T,Etrain%*%W[,i]) }

ey<-t(T[,i])%*%T[,i]

ey1<-as.numeric(ey)

if (i==1) {

P[,i]<-t(Etrain)%*%T[,i]/ey1

Q[i]<-t(Ftrain)%*%T[,i]/ey1 }

else {

P<-cbind(P,t(Etrain)%*%T[,i]/ey1)

Q<-cbind(Q,t(Ftrain)%*%T[,i]/ey1) }

Etrain<-Etrain-T[,i]%*%t(P[,i])

Ftrain<-Ftrain-T[,i]%*%t(Q[i])

A<-solve(t(T)%*%T)%*%t(T)%*%lj

R<-W%*%solve(t(P)%*%W)

B<-R%*%A

B.pred[i,m]<-Xb%*%B+t(t(mean(Btrain)))

}

}

for (i in 1:ncomp) {

PRESS[i]<-0}

for (i in 1:ncomp) {

for (m in 1:nrow(Fe)) {

PRESS[i]<-PRESS[i]+(Btest[m]-B.pred[i,m])^2}

RMSECV[i]<-sqrt(PRESS[i]/(nrow(Fe)))}

RMSECV

ncomp<-which.min(RMSECV)

BB<- matrix(rep(NA,nrow(Fe)*ncol(Fe)),nrow=nrow(Fe))

xtrain<-matrix(rep(NA, (nrow(Fe)-1)*ncol(Fe)),nrow=(nrow(Fe)-1))

for (i in 1:ncomp) {

PRESS[i]<-0}

for (m in 1:nrow(Fe)) {

xtrain<-Fe[-m,]

Btrain<-B1[-m]

Etrain<-t(t(xtrain)-colMeans(xtrain))

Ftrain<-Btrain-mean(Btrain)

lj<-Ftrain

T<-matrix(rep(NA, nrow(Etrain)*1),nrow=nrow(Etrain))

W<-matrix(rep(NA, ncol(Etrain)*1),nrow=ncol(Etrain))

P<-matrix(rep(NA, ncol(Etrain)*1),nrow=ncol(Etrain))

Q<-array(1)

for (i in 1:ncomp) {

S<-t(Etrain)%*%Ftrain

qp<-svd(S)

if (i==1) {

W[,i]<-qp$u

T[,i]<-Etrain%*%W[,i] }

else {

W<-cbind(W,qp$u)

T<-cbind(T,Etrain%*%W[,i]) }

ey<-t(T[,i])%*%T[,i]

ey1<-as.numeric(ey)

if (i==1) {

P[,i]<-t(Etrain)%*%T[,i]/ey1

Q[i]<-t(Ftrain)%*%T[,i]/ey1 }

else {

P<-cbind(P,t(Etrain)%*%T[,i]/ey1)

Q<-cbind(Q,t(Ftrain)%*%T[,i]/ey1) }

Etrain<-Etrain-T[,i]%*%t(P[,i])

Ftrain<-Ftrain-T[,i]%*%t(Q[i])

A<-solve(t(T)%*%T)%*%t(T)%*%lj

R<-W%*%solve(t(P)%*%W)

}

B<-R%*%A

BB[m,]<-t(B)

for (i in 1:ncol(BB)) {

BB[m,i]<-abs(BB[m,i])}

skala<-BB[m,which.max(BB[m,])]

BB[m,]<-BB[m,]/skala

}

ploz<-array(pon)

ploz1<-array(pon)

Feg<-F

dim(F)

Stdev<-array(ncol(BB))

Me<-array(ncol(BB))

Tvalue<-array(ncol(BB))

for (i in 1:ncol(BB)) {

Me[i]<-mean(BB[,i])

Stdev[i]<-0

for (j in 1:nrow(BB)) {

Stdev[i]<-Stdev[i]+((BB[j,i]-Me[i])^2)}

Stdev[i]<-sqrt(Stdev[i]/(nrow(BB)-1))

Tvalue[i]<-Me[i]/Stdev[i]}

Ton<-Tvalue

for (i in 1:(ncomp+2)) {

Ton[which.max(Ton)]<-0}

pon<-trunc(Ton[which.max(Ton)])*2

for (h in 1:pon) {

brojac<-0

Zerd<-c()

Bvar<-c()

for (i in 1:ncol(Feg)) {

if (Tvalue[i]>(h*0.5)) {

brojac<-brojac+1

if (brojac==1) {

Zerd<-Feg[,i]

Bvar<-i}

else {

Zerd<-cbind(Zerd,Feg[,i])

Bvar<-cbind(Bvar,i)}}

}

Bvar<-as.vector(Bvar)

F<-Zerd

V<-data.frame(F=I(F),**B2=B2**)

Vtest<-seq(sekta,length(B2),by=5)

Vrum<-seq(1,length(B2),by=1)

Vtrain<-Vrum[-Vtest]

B1<-V$B2[Vtrain]

Fe<-V$F[Vtrain,]

B.predd<-matrix(rep(NA, ncomp*nrow(Fe)),nrow=ncomp)

RMSECV<-array(ncomp)

PRESS<-array(ncomp)

Btest<-array(nrow(Fe))

xtrain<-Fe

xtest<-matrix(rep(NA,1*ncol(Fe)),nrow=1)

B.pred<-matrix(rep(NA, ncomp*nrow(Fe)),nrow=ncomp)

for (i in 1:ncomp) {

PRESS[i]<-0}

for (m in 1:nrow(Fe)) {

xtest<-Fe[m,]

Btest[m]<-B1[m]

xtrain<-Fe[-m,]

Btrain<-B1[-m]

Etrain<-t(t(xtrain)-colMeans(xtrain))

Xb<-xtest- colMeans(xtrain)

Ftrain<-Btrain-mean(Btrain)

lj<-Ftrain

T<-matrix(rep(NA, nrow(Etrain)*1),nrow=nrow(Etrain))

W<-matrix(rep(NA, ncol(Etrain)*1),nrow=ncol(Etrain))

P<-matrix(rep(NA, ncol(Etrain)*1),nrow=ncol(Etrain))

Q<-array(1)

for (i in 1:ncomp) {

S<-t(Etrain)%*%Ftrain

qp<-svd(S)

if (i==1) {

W[,i]<-qp$u

T[,i]<-Etrain%*%W[,i] }

else {

W<-cbind(W,qp$u)

T<-cbind(T,Etrain%*%W[,i]) }

ey<-t(T[,i])%*%T[,i]

ey1<-as.numeric(ey)

if (i==1) {

P[,i]<-t(Etrain)%*%T[,i]/ey1

Q[i]<-t(Ftrain)%*%T[,i]/ey1 }

else {

P<-cbind(P,t(Etrain)%*%T[,i]/ey1)

Q<-cbind(Q,t(Ftrain)%*%T[,i]/ey1) }

Etrain<-Etrain-T[,i]%*%t(P[,i])

Ftrain<-Ftrain-T[,i]%*%t(Q[i])

A<-solve(t(T)%*%T)%*%t(T)%*%lj

R<-W%*%solve(t(P)%*%W)

B<-R%*%A

B.pred[i,m]<-Xb%*%B+t(t(mean(Btrain)))

}

}

for (i in 1:ncomp) {

for (m in 1:nrow(Fe)) {

PRESS[i]<-PRESS[i]+(Btest[m]-B.pred[i,m])^2}

RMSECV[i]<-sqrt(PRESS[i]/(nrow(Fe)))}

ploz[h]<-RMSECV[which.min(RMSECV)]

}

h<-which.min(ploz)

brojac<-0

Zerd<-c()

Bvar<-c()

for (i in 1:ncol(Feg)) {

if (Tvalue[i]>(h*0.5)) {

brojac<-brojac+1

if (brojac==1) {

Zerd<-Feg[,i]

Bvar<-i}

else {

Zerd<-cbind(Zerd,Feg[,i])

Bvar<-cbind(Bvar,i)}}

}

Bvar<-as.vector(Bvar)

F<-Zerd

dim(F)

V<-data.frame(F=I(F),**B2=B2**)

Vtest<-seq(sekta,length(B2),by=5)

Vrum<-seq(1,length(B2),by=1)

Vtrain<-Vrum[-Vtest]

B1<-V$B2[Vtrain]

Fe<-V$F[Vtrain,]

B.predd<-matrix(rep(NA, ncomp*nrow(Fe)),nrow=ncomp)

RMSECV<-array(ncomp)

PRESS<-array(ncomp)

Btest<-array(nrow(Fe))

xtrain<-Fe

xtest<-matrix(rep(NA,1*ncol(Fe)),nrow=1)

B.pred<-matrix(rep(NA, ncomp*nrow(Fe)),nrow=ncomp)

for (i in 1:ncomp) {

PRESS[i]<-0}

for (m in 1:nrow(Fe)) {

xtest<-Fe[m,]

Btest[m]<-B1[m]

xtrain<-Fe[-m,]

Btrain<-B1[-m]

Etrain<-t(t(xtrain)-colMeans(xtrain))

Xb<-xtest- colMeans(xtrain)

Ftrain<-Btrain-mean(Btrain)

lj<-Ftrain

T<-matrix(rep(NA, nrow(Etrain)*1),nrow=nrow(Etrain))

W<-matrix(rep(NA, ncol(Etrain)*1),nrow=ncol(Etrain))

P<-matrix(rep(NA, ncol(Etrain)*1),nrow=ncol(Etrain))

Q<-array(1)

for (i in 1:ncomp) {

S<-t(Etrain)%*%Ftrain

qp<-svd(S)

if (i==1) {

W[,i]<-qp$u

T[,i]<-Etrain%*%W[,i] }

else {

W<-cbind(W,qp$u)

T<-cbind(T,Etrain%*%W[,i]) }

ey<-t(T[,i])%*%T[,i]

ey1<-as.numeric(ey)

if (i==1) {

P[,i]<-t(Etrain)%*%T[,i]/ey1

Q[i]<-t(Ftrain)%*%T[,i]/ey1 }

else {

P<-cbind(P,t(Etrain)%*%T[,i]/ey1)

Q<-cbind(Q,t(Ftrain)%*%T[,i]/ey1) }

Etrain<-Etrain-T[,i]%*%t(P[,i])

Ftrain<-Ftrain-T[,i]%*%t(Q[i])

A<-solve(t(T)%*%T)%*%t(T)%*%lj

R<-W%*%solve(t(P)%*%W)

B<-R%*%A

B.pred[i,m]<-Xb%*%B+t(t(mean(Btrain)))

}

}

for (i in 1:ncomp) {

for (m in 1:nrow(Fe)) {

PRESS[i]<-PRESS[i]+(Btest[m]-B.pred[i,m])^2}

RMSECV[i]<-sqrt(PRESS[i]/(nrow(Fe)))}

ncomp<-which.min(RMSECV)

xtrain<-V$F[Vtrain,]

xtest<-V$F[Vtest,]

Btrain<-V$B2[Vtrain]

Btest<-V$B2[Vtest]

X1<-mean(Btrain)

Ftrain<-array(length(Btrain))

for (i in 1:length(Btrain)) {

Ftrain[i]<-Btrain[i]-X1}

X<-colMeans(xtrain)

for (i in 1:nrow(xtrain)) {

if (i==1) {

X2<-X}

else {

X2<-rbind(X2,X) }}

Etrain<-xtrain-X2

Etr<-Etrain

for (hj in 1:length(Btest)) {

if (hj==1) {

X2<-X }

else {

X2<-rbind(X2,X)}}

Xb<-xtest-X2

lj<-Ftrain

T<-matrix(rep(NA, nrow(Etrain)*1),nrow=nrow(Etrain))

W<-matrix(rep(NA, ncol(Etrain)*1),nrow=ncol(Etrain))

P<-matrix(rep(NA, ncol(Etrain)*1),nrow=ncol(Etrain))

Q<-array(1)

for (i in 1:ncomp) {

S<-t(Etrain)%*%Ftrain

qp<-svd(S)

if (i==1) {

W[,i]<-qp$u

T[,i]<-Etrain%*%W[,i] }

else {

W<-cbind(W,qp$u)

T<-cbind(T,Etrain%*%W[,i]) }

ey<-t(T[,i])%*%T[,i]

ey1<-as.numeric(ey)

if (i==1) {

P[,i]<-t(Etrain)%*%T[,i]/ey1

Q[i]<-t(Ftrain)%*%T[,i]/ey1 }

else {

P<-cbind(P,t(Etrain)%*%T[,i]/ey1)

Q<-cbind(Q,t(Ftrain)%*%T[,i]/ey1) }

Etrain<-Etrain-T[,i]%*%t(P[,i])

Ftrain<-Ftrain-T[,i]%*%t(Q[i])

}

A<-solve(t(T)%*%T)%*%t(T)%*%lj

R<-W%*%solve(t(P)%*%W)

B<-R%*%A

B.pred.train<-array(length(Btrain))

B.pred.test<-array(length(Btest))

B.predd.train<-array(length(Btrain))

B.predd.test<-array(length(Btest))

PCCtrain<-0

PCCtest<-0

for (i in 1:length(Btrain)) {

B.pred.train[i]<-Etr[i,]%*%B+t(t(X1))

if (B.pred.train[i]>0.5) {

B.predd.train[i]<-1}

else {

B.predd.train[i]<-0}

if (B.predd.train[i]==Btrain[i]) {

PCCtrain<-PCCtrain+1/length(Btrain)}

}

for (i in 1:length(Btest)) {

B.pred.test[i]<-Xb[i,]%*%B+t(t(X1))

if (B.pred.test[i]>0.5) {

B.predd.test[i]<-1}

else {

B.predd.test[i]<-0}

if (B.predd.test[i]==Btest[i]) {

PCCtest<-PCCtest+1/length(Btest)}

}

ncomp

RMSEC<-sqrt(sum((B.pred.train-Btrain)^2)/(nrow(Fe)-1-ncomp))

RMSEP<-sqrt(sum((B.pred.test-Btest)^2)/(length(Btest)))

k1<-B.pred.train

k<-Btrain

a<-array(length(k1))

for (i in 1:length(k1)) {

a[i]<-k1[i]}

k1<-a

L<-length(k)

e<-matrix(rep(NA,2,nrow=2))

x1<- matrix(rep(NA, L*2),nrow= L)

y1<- matrix(rep(NA, L),nrow= L)

for (i in 1:length(k)) {

for(j in 1:2) {

x1[i,j]<-k1[i]^(j-1)

}

}

z<-solve(t(x1)%*%x1)%*%t(x1)

for (i in 1:length(k1)) {

y1[i,1]<-k[i]

}

e<-z%*%y1

w<-array(length(k))

for (i in 1:length(k)) {

w[i]<-(x1[i,2]*e[2,1])+e[1,1] }

w

f<-0

for (i in 1:length(k)) {

f<-f+y1[i,1]}

f<-f/length(k)

x11<-matrix(rep(NA, length(k)*2),nrow= length(k))

for (i11 in 1:length(k)) {

for(j11 in 1:2) {

x11[i11,j11]<-w[i11]^(j11-1)}

}

z11<-solve(t(x11)%*%x11)%*%t(x11)

e11<-z11%*%y1

Ttest.pred2<-array(length(k))

for (i in 1:length(k)) {

Ttest.pred2[i]<-(x11[i,2]*e11[2,1])+e11[1,1] }

g<-0

g1<-0

for (i in 1:length(k)) {

g<-g+(y1[i,1]-f)*(y1[i,1]-f)

g1<-g1+(y1[i,1]-Ttest.pred2[i])*(y1[i,1]-Ttest.pred2[i]) }

r2tr<-1-(g1/g)

r2tr

k1<-B.pred.test

k<-Btest

a<-array(length(k1))

for (i in 1:length(k1)) {

a[i]<-k1[i]}

k1<-a

L<-length(k)

e<-matrix(rep(NA,2,nrow=2))

x1<- matrix(rep(NA, L*2),nrow= L)

y1<- matrix(rep(NA, L),nrow= L)

for (i in 1:length(k)) {

for(j in 1:2) {

x1[i,j]<-k1[i]^(j-1)

}

}

z<-solve(t(x1)%*%x1)%*%t(x1)

for (i in 1:length(k1)) {

y1[i,1]<-k[i]

}

e<-z%*%y1

w<-array(length(k))

for (i in 1:length(k)) {

w[i]<-(x1[i,2]*e[2,1])+e[1,1] }

w

f<-0

for (i in 1:length(k)) {

f<-f+y1[i,1]}

f<-f/length(k)

x11<-matrix(rep(NA, length(k)*2),nrow= length(k))

for (i11 in 1:length(k)) {

for(j11 in 1:2) {

x11[i11,j11]<-w[i11]^(j11-1)}

}

z11<-solve(t(x11)%*%x11)%*%t(x11)

e11<-z11%*%y1

Ttest.pred2<-array(length(k))

for (i in 1:length(k)) {

Ttest.pred2[i]<-(x11[i,2]*e11[2,1])+e11[1,1] }

g<-0

g1<-0

for (i in 1:length(k)) {

g<-g+(y1[i,1]-f)*(y1[i,1]-f)

g1<-g1+(y1[i,1]-Ttest.pred2[i])*(y1[i,1]-Ttest.pred2[i]) }

r2te<-1-(g1/g)

r2te

ncomp

RMSEC

RMSEP

r2tr

r2te

RMSECV[which.min(RMSECV)]

B.pred.S<-array(nrow(Fe5))

Fe5<-Fe5[,Bvar]

for (i in 1:nrow(Fe5)) {

B.pred.S[i]<- Fe5[i,]%*%B+t(t(X1))}

B.pred.S

Labels<- read.table("**Labels_of_Sigma1_59_S_siramesine_similar_comp_from_GOLD_1D_2D_3D_flex_1836_var**.txt")

t(cbind(c('siramesine'),t(Labels)))

cbind(t(cbind(c('siramesine'),t(Labels))),t(t(B.pred.S))) #and compare with Gold pKi results in Table 4
