# Supplementary material for: In Vitro Confirmation of Siramesine as a Novel Antifungal Agent with In Silico Lead Proposals of Structurally Related Antifungals
Source: Molecules. 2021 Jun 8;26(12):3504. doi: 10.3390/molecules26123504 (PMC8230181; doi:10.3390/molecules26123504)
Supplement: Supplementary file 1 [file molecules-26-03504-s001.zip › Supplementary files/Supplementary_data_files.zip!/Supplementary_data_files/UVE_PLS_code_for_ERG2_samples_crossvalidation.doc]

A1<-read.table("B:\\Ozren\\Clanak_3_siramesine\\PLS\\ERG2_44_lit_uz_without_NaN_from_GOLD.txt")

B2<-c(5.7, 5.5, 7.19, 5.15, 4.78, 5, 7.21, 5.92, 6.8, 4.5, 9.3, 7.19, 10.3, 8.3, 9, 5.33, 6.3, 6.51, 4, 5.55, 9.15, 6.63, 9.4, 6, 6.55, 5.35, 7.77, 8.05, 7.89, 4.3, 5.82, 7.18, 4, 5.93, 5.11, 8.7, 5.72, 10.05, 6.3, 9.82, 9.7, 6.77, 8.7, 6.41)

pon<-150

ncomp<-20 #novo

Fe1<-data.matrix(A1)

F<-Fe1

Sys.time()

sekta<-3

V<-data.frame(F=I(F),**B2=B2**)

Vrum<-seq(1,length(B2),by=1)

Vtrain<-Vrum

B1<-V$B2[Vtrain]

Fe<-V$F[Vtrain,]

broelim<-0

for (i in 1:ncol(Fe)) {

if (length(unique(Fe[,i]))<3) {

broelim<-broelim+1

if (broelim==1)

vectvoid<-c(i)

else

vectvoid<-cbind(vectvoid,i)}

}

for (i in length(vectvoid):1) {

Fe<-Fe[,-vectvoid[i]]

F<-F[,-vectvoid[i]]}

A5<-read.table("B:\\Ozren\\Clanak_3_siramesine\\PLS\\ERG2_S_Siramesini_with_Siramesine_from_Gold_uskladjeno_s_ERG2_44_lit_without_NaN.txt")

Fe5<-data.matrix(A5)

for (i in length(vectvoid):1) {

Fe5<-Fe5[,-vectvoid[i]]}

Bvarr<-c()

minn<-array(ncol(F))

maxx<-array(ncol(F))

for (i in 1:ncol(F)) {

minn[i]<-min(F[,i])

maxx[i]<-max(F[,i])

bro<-0

for (j in 1:nrow(Fe5)) {

if ((Fe5[j,i]>=minn[i])&&(Fe5[j,i]<=maxx[i]))

bro<-bro+1}

if (bro==nrow(Fe5))

Bvarr<-cbind(Bvarr,c(i))}

Fe<-Fe[,Bvarr]

F<-F[,Bvarr]

Fe5<-Fe5[,Bvarr]

meanvect<-array(ncol(Fe))

sdvect<-array(ncol(Fe))

for (i in 1:ncol(Fe)) {

meanvect[i]<-mean(Fe[,i])

sdvect[i]<-sd(Fe[,i])}

Fe<-scale(Fe) #SVE POD CRVENOM mora ići za #skaliranje ILI ništa!

for (i in 1:ncol(F)) {

F[,i]<- (F[,i]- meanvect[i])/sdvect[i] }

for (i in 1:ncol(Fe5)) {

Fe5[,i]<- (Fe5[,i]- meanvect[i])/sdvect[i] }

V<-data.frame(F=I(F),**B2=B2**)

Vrum<-seq(1,length(B2),by=1)

Vtrain<-Vrum

B1<-V$B2[Vtrain]

Fe<-V$F[Vtrain,]

RMSECV<-array(ncomp)

PRESS<-array(ncomp)

Btest<-array(nrow(Fe))

xtrain<-Fe

xtest<-matrix(rep(NA,1*ncol(Fe)),nrow=1)

B.pred<-matrix(rep(NA, ncomp*nrow(Fe)),nrow=ncomp)

for (i in 1:ncomp) {

PRESS[i]<-0}

for (m in 1:nrow(Fe)) {

xtest<-Fe[m,]

Btest[m]<-B1[m]

xtrain<-Fe[-m,]

Btrain<-B1[-m]

Ftrain<-t(t(Btrain)-mean(Btrain))

Etrain<-t(t(xtrain)-colMeans(xtrain))

Xb<-xtest- colMeans(xtrain)

lj<-Ftrain

T<-matrix(rep(NA, nrow(Etrain)*1),nrow=nrow(Etrain))

W<-matrix(rep(NA, ncol(Etrain)*1),nrow=ncol(Etrain))

P<-matrix(rep(NA, ncol(Etrain)*1),nrow=ncol(Etrain))

Q<-array(1)

for (i in 1:ncomp) {

S<-t(Etrain)%*%Ftrain

qp<-svd(S)

if (i==1) {

W[,i]<-qp$u

T[,i]<-Etrain%*%W[,i] }

else {

W<-cbind(W,qp$u)

T<-cbind(T,Etrain%*%W[,i]) }

ey<-t(T[,i])%*%T[,i]

ey1<-as.numeric(ey)

if (i==1) {

P[,i]<-t(Etrain)%*%T[,i]/ey1

Q[i]<-t(Ftrain)%*%T[,i]/ey1 }

else {

P<-cbind(P,t(Etrain)%*%T[,i]/ey1)

Q<-cbind(Q,t(Ftrain)%*%T[,i]/ey1) }

Etrain<-Etrain-T[,i]%*%t(P[,i])

Ftrain<-Ftrain-T[,i]%*%t(Q[i])

A<-solve(t(T)%*%T)%*%t(T)%*%lj

R<-W%*%solve(t(P)%*%W)

B<-R%*%A

B.pred[i,m]<-Xb%*%B+t(t(mean(Btrain)))

}

}

for (i in 1:ncomp) {

PRESS[i]<-0}

for (i in 1:ncomp) {

for (m in 1:nrow(Fe)) {

PRESS[i]<-PRESS[i]+(Btest[m]-B.pred[i,m])^2}

RMSECV[i]<-sqrt(PRESS[i]/(nrow(Fe)))}

RMSECV

ncomp<-which.min(RMSECV)

BB<- matrix(rep(NA,nrow(Fe)*ncol(Fe)),nrow=nrow(Fe))

xtrain<-matrix(rep(NA, (nrow(Fe)-1)*ncol(Fe)),nrow=(nrow(Fe)-1))

for (i in 1:ncomp) {

PRESS[i]<-0}

for (m in 1:nrow(Fe)) {

xtrain<-Fe[-m,]

Btrain<-B1[-m]

Etrain<-t(t(xtrain)-colMeans(xtrain))

Ftrain<-Btrain-mean(Btrain)

lj<-Ftrain

T<-matrix(rep(NA, nrow(Etrain)*1),nrow=nrow(Etrain))

W<-matrix(rep(NA, ncol(Etrain)*1),nrow=ncol(Etrain))

P<-matrix(rep(NA, ncol(Etrain)*1),nrow=ncol(Etrain))

Q<-array(1)

for (i in 1:ncomp) {

S<-t(Etrain)%*%Ftrain

qp<-svd(S)

if (i==1) {

W[,i]<-qp$u

T[,i]<-Etrain%*%W[,i] }

else {

W<-cbind(W,qp$u)

T<-cbind(T,Etrain%*%W[,i]) }

ey<-t(T[,i])%*%T[,i]

ey1<-as.numeric(ey)

if (i==1) {

P[,i]<-t(Etrain)%*%T[,i]/ey1

Q[i]<-t(Ftrain)%*%T[,i]/ey1 }

else {

P<-cbind(P,t(Etrain)%*%T[,i]/ey1)

Q<-cbind(Q,t(Ftrain)%*%T[,i]/ey1) }

Etrain<-Etrain-T[,i]%*%t(P[,i])

Ftrain<-Ftrain-T[,i]%*%t(Q[i])

A<-solve(t(T)%*%T)%*%t(T)%*%lj

R<-W%*%solve(t(P)%*%W)

}

B<-R%*%A

BB[m,]<-t(B)

for (i in 1:ncol(BB)) {

BB[m,i]<-abs(BB[m,i])}

skala<-BB[m,which.max(BB[m,])]

BB[m,]<-BB[m,]/skala

}

ploz<-array(pon)

ploz1<-array(pon)

Feg<-F

dim(F)

Stdev<-array(ncol(BB))

Me<-array(ncol(BB))

Tvalue<-array(ncol(BB))

for (i in 1:ncol(BB)) {

Me[i]<-mean(BB[,i])

Stdev[i]<-0

for (j in 1:nrow(BB)) {

Stdev[i]<-Stdev[i]+((BB[j,i]-Me[i])^2)}

Stdev[i]<-sqrt(Stdev[i]/(nrow(BB)-1))

Tvalue[i]<-Me[i]/Stdev[i]}

Ton<-Tvalue

for (i in 1:(ncomp+2)) {

Ton[which.max(Ton)]<-0}

pon<-trunc(Ton[which.max(Ton)])*2

for (h in 1:pon) {

brojac<-0

Zerd<-c()

Bvar<-c()

for (i in 1:ncol(Feg)) {

if (Tvalue[i]>(h*0.5)) {

brojac<-brojac+1

if (brojac==1) {

Zerd<-Feg[,i]

Bvar<-i}

else {

Zerd<-cbind(Zerd,Feg[,i])

Bvar<-cbind(Bvar,i)}}

}

Bvar<-as.vector(Bvar)

F<-Zerd

V<-data.frame(F=I(F),**B2=B2**)

Vrum<-seq(1,length(B2),by=1)

Vtrain<-Vrum

B1<-V$B2[Vtrain]

Fe<-V$F[Vtrain,]

B.predd<-matrix(rep(NA, ncomp*nrow(Fe)),nrow=ncomp)

RMSECV<-array(ncomp)

PRESS<-array(ncomp)

Btest<-array(nrow(Fe))

xtrain<-Fe

xtest<-matrix(rep(NA,1*ncol(Fe)),nrow=1)

B.pred<-matrix(rep(NA, ncomp*nrow(Fe)),nrow=ncomp)

for (i in 1:ncomp) {

PRESS[i]<-0}

for (m in 1:nrow(Fe)) {

xtest<-Fe[m,]

Btest[m]<-B1[m]

xtrain<-Fe[-m,]

Btrain<-B1[-m]

Etrain<-t(t(xtrain)-colMeans(xtrain))

Xb<-xtest- colMeans(xtrain)

Ftrain<-Btrain-mean(Btrain)

lj<-Ftrain

T<-matrix(rep(NA, nrow(Etrain)*1),nrow=nrow(Etrain))

W<-matrix(rep(NA, ncol(Etrain)*1),nrow=ncol(Etrain))

P<-matrix(rep(NA, ncol(Etrain)*1),nrow=ncol(Etrain))

Q<-array(1)

for (i in 1:ncomp) {

S<-t(Etrain)%*%Ftrain

qp<-svd(S)

if (i==1) {

W[,i]<-qp$u

T[,i]<-Etrain%*%W[,i] }

else {

W<-cbind(W,qp$u)

T<-cbind(T,Etrain%*%W[,i]) }

ey<-t(T[,i])%*%T[,i]

ey1<-as.numeric(ey)

if (i==1) {

P[,i]<-t(Etrain)%*%T[,i]/ey1

Q[i]<-t(Ftrain)%*%T[,i]/ey1 }

else {

P<-cbind(P,t(Etrain)%*%T[,i]/ey1)

Q<-cbind(Q,t(Ftrain)%*%T[,i]/ey1) }

Etrain<-Etrain-T[,i]%*%t(P[,i])

Ftrain<-Ftrain-T[,i]%*%t(Q[i])

A<-solve(t(T)%*%T)%*%t(T)%*%lj

R<-W%*%solve(t(P)%*%W)

B<-R%*%A

B.pred[i,m]<-Xb%*%B+t(t(mean(Btrain)))

}

}

for (i in 1:ncomp) {

for (m in 1:nrow(Fe)) {

PRESS[i]<-PRESS[i]+(Btest[m]-B.pred[i,m])^2}

RMSECV[i]<-sqrt(PRESS[i]/(nrow(Fe)))}

ploz[h]<-RMSECV[which.min(RMSECV)]

}

h<-which.min(ploz)

h<-18 # due to the restriction of maximum number of descriptors (200)

brojac<-0

Zerd<-c()

Bvar<-c()

for (i in 1:ncol(Feg)) {

if (Tvalue[i]>(h*0.5)) {

brojac<-brojac+1

if (brojac==1) {

Zerd<-Feg[,i]

Bvar<-i}

else {

Zerd<-cbind(Zerd,Feg[,i])

Bvar<-cbind(Bvar,i)}}

}

Bvar<-as.vector(Bvar)

F<-Zerd

dim(F)

V<-data.frame(F=I(F),**B2=B2**)

Vrum<-seq(1,length(B2),by=1)

Vtrain<-Vrum

B1<-V$B2[Vtrain]

Fe<-V$F[Vtrain,]

B.predd<-matrix(rep(NA, ncomp*nrow(Fe)),nrow=ncomp)

RMSECV<-array(ncomp)

PRESS<-array(ncomp)

Btest<-array(nrow(Fe))

xtrain<-Fe

xtest<-matrix(rep(NA,1*ncol(Fe)),nrow=1)

B.pred<-matrix(rep(NA, ncomp*nrow(Fe)),nrow=ncomp)

for (i in 1:ncomp) {

PRESS[i]<-0}

for (m in 1:nrow(Fe)) {

xtest<-Fe[m,]

Btest[m]<-B1[m]

xtrain<-Fe[-m,]

Btrain<-B1[-m]

Etrain<-t(t(xtrain)-colMeans(xtrain))

Xb<-xtest- colMeans(xtrain)

Ftrain<-Btrain-mean(Btrain)

lj<-Ftrain

T<-matrix(rep(NA, nrow(Etrain)*1),nrow=nrow(Etrain))

W<-matrix(rep(NA, ncol(Etrain)*1),nrow=ncol(Etrain))

P<-matrix(rep(NA, ncol(Etrain)*1),nrow=ncol(Etrain))

Q<-array(1)

for (i in 1:ncomp) {

S<-t(Etrain)%*%Ftrain

qp<-svd(S)

if (i==1) {

W[,i]<-qp$u

T[,i]<-Etrain%*%W[,i] }

else {

W<-cbind(W,qp$u)

T<-cbind(T,Etrain%*%W[,i]) }

ey<-t(T[,i])%*%T[,i]

ey1<-as.numeric(ey)

if (i==1) {

P[,i]<-t(Etrain)%*%T[,i]/ey1

Q[i]<-t(Ftrain)%*%T[,i]/ey1 }

else {

P<-cbind(P,t(Etrain)%*%T[,i]/ey1)

Q<-cbind(Q,t(Ftrain)%*%T[,i]/ey1) }

Etrain<-Etrain-T[,i]%*%t(P[,i])

Ftrain<-Ftrain-T[,i]%*%t(Q[i])

A<-solve(t(T)%*%T)%*%t(T)%*%lj

R<-W%*%solve(t(P)%*%W)

B<-R%*%A

B.pred[i,m]<-Xb%*%B+t(t(mean(Btrain)))

}

}

for (i in 1:ncomp) {

for (m in 1:nrow(Fe)) {

PRESS[i]<-PRESS[i]+(Btest[m]-B.pred[i,m])^2}

RMSECV[i]<-sqrt(PRESS[i]/(nrow(Fe)))}

ncomp<-which.min(RMSECV)

xtrain<-V$F[Vtrain,]

Btrain<-V$B2[Vtrain]

X1<-mean(Btrain)

Ftrain<-array(length(Btrain))

for (i in 1:length(Btrain)) {

Ftrain[i]<-Btrain[i]-X1}

X<-colMeans(xtrain)

for (i in 1:nrow(xtrain)) {

if (i==1) {

X2<-X}

else {

X2<-rbind(X2,X) }}

Etrain<-xtrain-X2

Etr<-Etrain

lj<-Ftrain

T<-matrix(rep(NA, nrow(Etrain)*1),nrow=nrow(Etrain))

W<-matrix(rep(NA, ncol(Etrain)*1),nrow=ncol(Etrain))

P<-matrix(rep(NA, ncol(Etrain)*1),nrow=ncol(Etrain))

Q<-array(1)

for (i in 1:ncomp) {

S<-t(Etrain)%*%Ftrain

qp<-svd(S)

if (i==1) {

W[,i]<-qp$u

T[,i]<-Etrain%*%W[,i] }

else {

W<-cbind(W,qp$u)

T<-cbind(T,Etrain%*%W[,i]) }

ey<-t(T[,i])%*%T[,i]

ey1<-as.numeric(ey)

if (i==1) {

P[,i]<-t(Etrain)%*%T[,i]/ey1

Q[i]<-t(Ftrain)%*%T[,i]/ey1 }

else {

P<-cbind(P,t(Etrain)%*%T[,i]/ey1)

Q<-cbind(Q,t(Ftrain)%*%T[,i]/ey1) }

Etrain<-Etrain-T[,i]%*%t(P[,i])

Ftrain<-Ftrain-T[,i]%*%t(Q[i])

}

A<-solve(t(T)%*%T)%*%t(T)%*%lj

R<-W%*%solve(t(P)%*%W)

B<-R%*%A

B.pred.train<-array(length(Btrain))

B.predd.train<-array(length(Btrain))

for (i in 1:length(Btrain)) {

B.pred.train[i]<-Etr[i,]%*%B+t(t(X1))

}

ncomp

RMSEC<-sqrt(sum((B.pred.train-Btrain)^2)/(nrow(Fe)-1-ncomp))

k1<-B.pred.train

k<-Btrain

a<-array(length(k1))

for (i in 1:length(k1)) {

a[i]<-k1[i]}

k1<-a

L<-length(k)

e<-matrix(rep(NA,2,nrow=2))

x1<- matrix(rep(NA, L*2),nrow= L)

y1<- matrix(rep(NA, L),nrow= L)

for (i in 1:length(k)) {

for(j in 1:2) {

x1[i,j]<-k1[i]^(j-1)

}

}

z<-solve(t(x1)%*%x1)%*%t(x1)

for (i in 1:length(k1)) {

y1[i,1]<-k[i]

}

e<-z%*%y1

w<-array(length(k))

for (i in 1:length(k)) {

w[i]<-(x1[i,2]*e[2,1])+e[1,1] }

w

f<-0

for (i in 1:length(k)) {

f<-f+y1[i,1]}

f<-f/length(k)

x11<-matrix(rep(NA, length(k)*2),nrow= length(k))

for (i11 in 1:length(k)) {

for(j11 in 1:2) {

x11[i11,j11]<-w[i11]^(j11-1)}

}

z11<-solve(t(x11)%*%x11)%*%t(x11)

e11<-z11%*%y1

Ttest.pred2<-array(length(k))

for (i in 1:length(k)) {

Ttest.pred2[i]<-(x11[i,2]*e11[2,1])+e11[1,1] }

g<-0

g1<-0

for (i in 1:length(k)) {

g<-g+(y1[i,1]-f)*(y1[i,1]-f)

g1<-g1+(y1[i,1]-Ttest.pred2[i])*(y1[i,1]-Ttest.pred2[i]) }

r2tr<-1-(g1/g)

r2tr

k1<-B.pred[ncomp,]

k<-Btrain

a<-array(length(k1))

for (i in 1:length(k1)) {

a[i]<-k1[i]}

k1<-a

L<-length(k)

e<-matrix(rep(NA,2,nrow=2))

x1<- matrix(rep(NA, L*2),nrow= L)

y1<- matrix(rep(NA, L),nrow= L)

for (i in 1:length(k)) {

for(j in 1:2) {

x1[i,j]<-k1[i]^(j-1)

}

}

z<-solve(t(x1)%*%x1)%*%t(x1)

for (i in 1:length(k1)) {

y1[i,1]<-k[i]

}

e<-z%*%y1

w<-array(length(k))

for (i in 1:length(k)) {

w[i]<-(x1[i,2]*e[2,1])+e[1,1] }

w

f<-0

for (i in 1:length(k)) {

f<-f+y1[i,1]}

f<-f/length(k)

x11<-matrix(rep(NA, length(k)*2),nrow= length(k))

for (i11 in 1:length(k)) {

for(j11 in 1:2) {

x11[i11,j11]<-w[i11]^(j11-1)}

}

z11<-solve(t(x11)%*%x11)%*%t(x11)

e11<-z11%*%y1

Ttest.pred2<-array(length(k))

for (i in 1:length(k)) {

Ttest.pred2[i]<-(x11[i,2]*e11[2,1])+e11[1,1] }

g<-0

g1<-0

for (i in 1:length(k)) {

g<-g+(y1[i,1]-f)*(y1[i,1]-f)

g1<-g1+(y1[i,1]-Ttest.pred2[i])*(y1[i,1]-Ttest.pred2[i]) }

r2trCV<-1-(g1/g)

r2trCV

ncomp

RMSEC

RMSECV

r2tr

r2trCV

RMSECV[which.min(RMSECV)]

B.pred.S<-array(nrow(Fe5))

Fe5<-Fe5[,Bvar]

for (i in 1:nrow(Fe5)) {

B.pred.S[i]<- Fe5[i,]%*%B+t(t(X1))}

t(t(B.pred.S))

Labels<-c('S05','S17','S10','S02','S08','S20','S14','S24','S12','S01','S29','S30','S38','S28','S25','S36','S41','S47','S45','S39','S80','S48','siramesine')

cbind(t(t(Labels)),t(t(B.pred.S)))
