# Supplementary material for: In Vitro Confirmation of Siramesine as a Novel Antifungal Agent with In Silico Lead Proposals of Structurally Related Antifungals
Source: Molecules. 2021 Jun 8;26(12):3504. doi: 10.3390/molecules26123504 (PMC8230181; doi:10.3390/molecules26123504)
Supplement: Supplementary file 1 [file molecules-26-03504-s001.zip › Supplementary files/Supplementary_information_list.doc]

3-PPP = 1200 x 10^-9 nM pKi 5.92

amiodarone = 62 x 10^-9 nM pKi 7.21

costicosterone (nema N) > 100000 x 10^-9 nM pKi 4

emopamil = 65 x 10^-9 nM pKi 7.19

enclomiphene = 160 x 10^-9 nM pKi 6.8

fenpropimorph = 0.05 x 10^-9 nM pKi 10.3

haloperidol = 0.5 x 10^-9 nM pKi 9.3

ifenprodil = 1 x 10^-9 nM pKi 9

MDL-28815 = 0.4 x 10^-9 nM pKi 9.4

pentazocine = 1000 x 10^-9 nM pKi 6

tamoxifene = 1500 x 10^-9 nM pKi 5.82

tridemorph = 0.09 x 10^-9 nM pKi 10.05

triparanol = 2 x 10^-9 nM pKi 8.7

zuclomiphene = 2 x 10^-9 nM pKi 8.7

nafoxidine = 232 x 10^-9 nM pKi 6.63

naftifine = 310 x 10^-9 nM pKi 6.51

ronipamil = 13 x 10^-9 nM pKi 7.89

SR-31747 = 9 x 10^-9 nM pKi 8.05

terbinafine > 50000 x 10^-9 nM pKi 4.3

trifluperidol = 0.15 x 10^-9 nM pKi 9.82

U-18666A = 0.2 x 10^-9 nM pKi 9.7

buflomedil = 7150 x 10^-9 nM pKi 5.15

carbisocaine = 2800 x 10^-9 nM pKi 5.55

raloxifene = 66 x 10^-9 nM pKi 7.18

tomatidine = 387 x 10^-9 nM pKi 6.41

solasodine = 1170 x 10^-9 nM pKi 5.93

solanidine > 100 000 x 10^-9 nM pKi 4

cyclopamine = 500 x 10^-9 nM pKi 6.3

jervine = 284 x 10^-9 nM pKi 6.55

1,3-di-o-tolylguanidine 2000 x 10^-9 nM pKi 5.7

AY-9944 = 65 x 10^-9 nM pKi 7.19

azidopamil = 10000 x 10^-9 nM pKi 5

BM-15766 = 16480 x 10^-9 nM pKi 4.78

CP-74932-4 = 31700 x 10^-9 nM pKi 4.50

L-690404 = 5 x 10^-9 nM pKi 8.3

MDL-5332 = 0.7 x 10^-9 nM pKi 9.15

N-Allylnormetazocine = 4730 x 10^-9 nM pKi 5.33

opipramol = 17 x 10^-9 nM pKi 7.77

progesterone = 4430 x 10^-9 nM pKi 5.35

R-59494 = 2800 x 10^-9 nM pKi 5.55

testosterone = 7760 x 10^-9 nM pKi 5.11

testosterone propionate = 1920 x 10^-9 nM pKi 5.72

trifluoperazine = 500 x 10^-9 nM pKi 6.3

MDL-5332 = 0.7 x 10^-9 nM pKi 9.15

VUF-8410 = 170 x 10^-9 nM pKi 6.77
