# Supplementary material for: In Vitro Confirmation of Siramesine as a Novel Antifungal Agent with In Silico Lead Proposals of Structurally Related Antifungals
Source: Molecules. 2021 Jun 8;26(12):3504. doi: 10.3390/molecules26123504 (PMC8230181; doi:10.3390/molecules26123504)
Supplement: Supplementary file 1 [file molecules-26-03504-s001.zip › Supplementary files/Supplementary_information_script_1_gromacs_Erg2_minim.doc]

grep -v HOH ERG2.pdb > ERG2_clean.pdb

gmx pdb2gmx -f ERG2_clean.pdb -o ERG2_processed.gro -water spce #15 OPLS SELECTED

gmx editconf -f ERG2_processed.gro -o ERG2_newbox.gro -c -d 1.0 -bt cubic

gmx solvate -cp ERG2_newbox.gro -cs spc216.gro -o ERG2_solv.gro -p topol.top

gmx grompp -f ions.mdp -c ERG2_solv.gro -p topol.top -o ions.tpr # USED mdtutorials.com/gmx/lysosyme/Files/ions.mdp

gmx genion -s ions.tpr -o ERG2_solv_ions.gro -p topol.top -pname NA -nname CL -neutral # 13 'SOL' SELECTED

gmx grompp -f em.mdp -c ERG2_solv_ions.gro -p topol.top -o em.tpr # <http://www.mdtutorials.com/gmx/lysozyme/Files/minim.mdp> USED

gmx mdrun -v -deffnm em
